# Assessing the role of conspiracy beliefs in oncological treatment decisions: An experimental approach

**DOI:** 10.1111/aphw.12615

**Published:** 2024-11-21

**Authors:** Florent Varet, Valentyn Fournier, Sylvain Delouvée

**Affiliations:** ^1^ ANTHROPO‐LAB ‐ ETHICS EA7446, Université Catholique de Lille, F‐59000 Lille, France; ^2^ Univ. Lille, CNRS, UMR 9193 ‐ SCALab ‐ Sciences Cognitives et Sciences Affectives Lille France; ^3^ LP3C (Laboratoire de Psychologie: Cognition, Comportement, Communication), Rennes 2 University Rennes France

**Keywords:** cancer, complementary and alternative medicine, conspiracy beliefs, conventional medicine, credibility, misinformation

## Abstract

Cancer is an important issue and a model topic for misinformatfion researchers. The present research experimentally investigates the effect of cancer‐related conspiracy beliefs and misinformation on oncology treatment intentions in a cancer‐free population. In three pre‐registered studies (*N* total = 1020), participants were asked to put themselves in the shoes of a patient recommended for chemotherapy. Study 1 (*N* = 300) failed to experimentally manipulate cancer‐related conspiracy beliefs with exposure to a health scandal not related to cancer. In Study 2 (*N* = 258), exposure to a pro‐conspiracy (vs. anti‐conspiracy) content related to cancer treatment was associated with more conspiracy beliefs, less intention to use chemotherapy and more intentions to use unconventional medicines. Exploratory analyses revealed that these effects were conditioned by the credibility of the misinformation. Study 3 (*N* = 462) replicated these findings using a full experimental design. Exposure (vs. no exposure) to a warning and accuracy prompt, prior to exposure to the pro‐conspiracy content, was found to be effective in reducing its credibility and preventing its detrimental effects. These findings corroborate the existence of an effect of conspiracy beliefs on treatment intentions in oncology and also suggest several ways to mitigate them.

## INTRODUCTION

Adherence to health recommendations is important in prevention and treatment of many diseases. Non‐adherence to medical recommendations may be driven by negative beliefs about these recommendations and healthcare actors, fuelled by misinformation and conspiracy beliefs (CBs) (e.g. Krastev et al., 2023; Natoli & Marques, 2021). Cancer is a leading cause of death worldwide, with approximately 20 million new cases, 9.7 million deaths in 2022, and about one in five people will develop cancer in their lifetime (World Health Organization, 2024). Among patients with cancer, adherence to conventional treatments (e.g. chemotherapy, radiation therapy) is an important predictor of survival and quality of life (Jacobs et al., 2019). As with non‐oncological treatments, adherence to oncological treatment may be impaired by negative beliefs about the treatment (e.g. concerns about side effects, low self‐efficacy, inadequate information) or health professionals (e.g. poor doctor‐patient relationship) (Lin et al., 2017; Toivonen et al., 2020). However, few empirical studies have investigated the potential impact of CBs and misinformation on treatment adherence in the oncology setting, despite cancer being a ‘model topic’ for misinformation research (Swire‐Thompson & Johnson, 2024). It is therefore crucial to investigate whether and to what extent CBs and misinformation influence treatment adherence in oncology.

### (Un)conventional medicines and CBs

Conventional medicine (also referred as ‘standard medical care’) refers to recognised treatments, accepted by medical experts as proper to treat diseases and widely used by healthcare professionals (National Cancer Institute, 2023). Complementary and alternative medicines (CAMs) encompass practices like mind–body therapies, naturopathy and energy medicine. Alternative medicines are used in replacement of conventional medicine whereas complementary medicines are used together with it (National Center for Complementary and Integrative Health, 2021). The use of CAM in patients with cancer is associated with impaired survival due to delayed initiation, poorer adherence or refusal of conventional treatments (Ben Kridis et al., 2021; Huiart et al., 2013; Johnson et al., 2018a,b). More broadly, preference for CAM may be associated with negative beliefs towards components or actors of conventional medicine (i.e. pharmaceutical industry) (Green et al., 2013), defiance towards the conventional healthcare system (Peterson et al., 2022), health‐related CBs (e.g. Galliford & Furnham, 2017; Soveri et al., 2021) and generic CBs (e.g. Lamberty & Imhoff, 2018).

CBs can be defined as proposed explanations of significant social and political events that identify a small group of people working in secret for their own benefit to the detriment of the public interest (Douglas et al., 2019). CBs, mistrust in authorities and preference for CAM may be linked by an overarching worldview that questions the morality of official authorities and denies science (Franks et al., 2017). A major vector of these beliefs is online misinformation, which refers to any online information contrary to current scientific data (Swire‐Thompson & Johnson, 2024).

### Cancer‐related misinformation and CBs

In oncology, the prevalence of online misinformation and its potential harm are considerable. A narrative review of the literature (Swire‐Thompson & Johnson, 2024) found that 11% to 100% of the top cancer‐related social media content contain misinformation. Johnson et al. (2022) found that 32.5% of the 200 most popular social media articles on the four most common cancers contained misinformation, including misleading descriptions, mischaracterizations of evidence strength or support for unproven therapies. Online cancer misinformation is likely to convey allegations of conspiracy against the health authorities, distrust in science and conventional medicine and preference for CAM (Grimes, 2022). The high prevalence of misinformation on the internet represents a great issue given that, despite healthcare professional remains the main source of information, internet arrives as a growingly common source of information in patients with cancer (Finney Rutten et al., 2016). According to Braun et al. (2019), 94% of cancer patients seek information related to their disease on the internet. Although seeking information online can have positive outcomes, exposure to misinformation can lead to misunderstandings or anxio‐depressive symptoms (for review, see Lleras de Frutos et al., 2020).

In addition to studies that have investigated the prevalence of online cancer misinformation, Fournier and Varet (2024) examined the associations between CBs and intention to use treatments among cancer‐free participants through a vignette methodology. The results corroborate findings showing that CBs can be harmful for patients with cancer. However, the cross‐sectional design of these studies did not allow a causal effect of CBs on health intentions to be tested.

## OVERVIEW

The present research relies on three studies aiming at experimentally testing the effect of CBs, conveyed by exposure to online misinformation, on the intention to use conventional cancer medicine and CAM. As in Fournier and Varet (2024), a cancer‐free population and a projection task (i.e. vignette design) were chosen to avoid causing patients a potential harm (Bradbury‐Jones et al., 2014). Study 1 was carried out to experimentally manipulate cancer treatment‐related CBs by exposing participants to a real non‐cancer‐related health scandal. Study 2 used an exposure to a fictitious scandal directly related to cancer treatment. Eventually, Study 3 was conducted to manipulate the credibility of the same scandal and assess a brief intervention to prevent the harm of misinformation.

**FIGURE 1:**
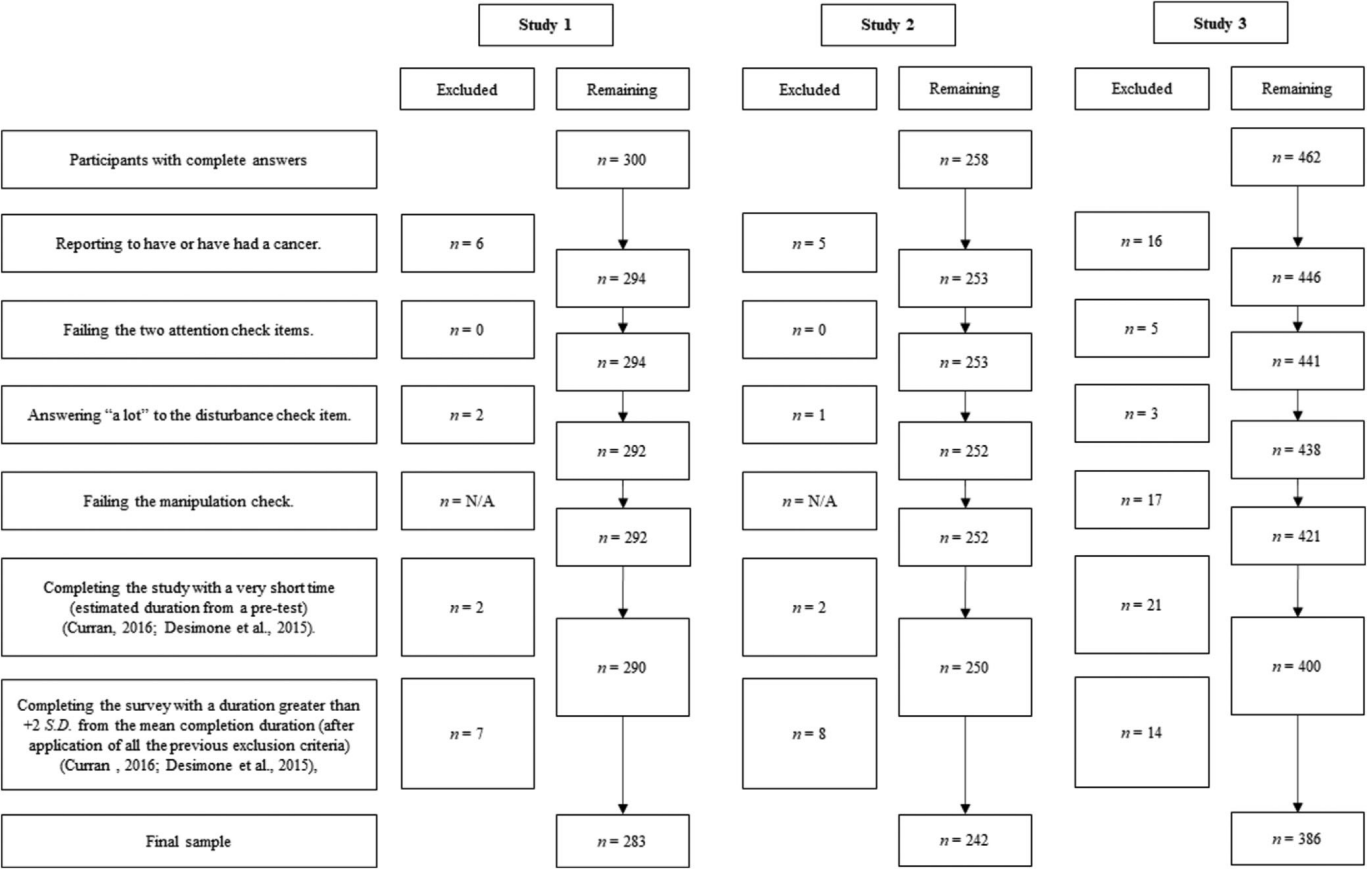
Flow chart specifying the exclusion criteria applied to participants in each study. N/A = not applicable. In Study 1, very short duration was <4 min 5 s in the Control condition or 5 min 35 s in the Conspiracy condition. Very long duration was >24 min 37 s in the Control condition or 30 min 14 s in the Conspiracy condition. In Study 2, very short duration was <4 min 05 s in both conditions. Very long duration was >24 min 37 s in both conditions. In Study 3, very short duration was <4 min 30s in the Control condition, 5 min 49 s in the Conspiracy condition or 6 min 18 s in the Prevention condition. Very long duration was >6 min 41 s in the Control condition, 27 min 43 s in the Conspiracy condition or 25 min 23 s in the Prevention condition.

All studies were conducted online with LimeSurvey. They were presented as investigating opinions on various societal issues, as well as knowledge and beliefs related to medicine and cancer treatments. All participants gave their informed consent. At the end of each study, participants were offered a debrief detailing the actual aims of the study and the fictitious nature of the conspiracy elements presented (for Study 2 & Study 3) and were invited to consult various reference resources on cancer management and the effects of chemotherapy. All studies were pre‐registered and priori power analyses computed (see Table 1). The exclusion criteria applied to participants in each study are presented in Figure 1. Most of confirmatory analyses planned in the pre‐registration forms were presented in this manuscript, while the others are presented in ‘Study details’ and ‘Deviations from pre‐registrations’ files. None of the exploratory analyses presented in this manuscript were pre‐registered, except for Study 3. Additional exploratory analyses are also presented in ‘Study details’ file. All multi‐item scales or subscales were averaged. All statistical analyses were carried out using Jamovi software, except for mediation analyses. These were carried out using PROCESS v.4.0. macro for SPSS, with an estimation of effect sizes and confidence intervals based on bootstrapping, 95% confidence and 5000 bootstrap samples for percentile bootstrap confidence intervals (default values) (Hayes, 2022). Pre‐registration and ‘Deviations from pre‐registrations’, ‘Study details’, ‘Materials’ and datasets are available in the Open Science Framework (OSF) repository (https://doi.org/10.17605/OSF.IO/2EDT9).

**TABLE 1:** Participant details for each study.

	Study 1	Study 2	Study 3
Online recruitment platform	Prolific	Prolific	Foule Factory
Country of location	France	France Belgium Luxembourg	France
*N* targeted in pre‐registration	232	232	348
*N* by condition	Control: 137 Conspiracy: 146	Pro‐conspiracy: 119 Anti‐conspiracy: 123	Control: 132 Conspiracy: 136 Prevention: 118
*M*_age_ (SD)	29.02 (8.88)	31.07 (9.90)	44.46 (12.49)
Gender	144 women 138 men 1 non‐binary	114 women 124 men 4 non‐binaries	194 women 192 men 0 non‐binaries
Socio‐professional categories	30% students, 6% unemployed, 17% employees or workers, 8% intermediate occupations, 5% tradespersons, shop or business owners or farmers, 33% executives or intellectual professions	25% students, 7% unemployed, 17% employees or workers, 13% intermediate occupations, 7% tradespersons, shop or business owners or farmers, 31% executives or intellectual professions	3% students, 13% unemployed, 35% employees or workers, 15% intermediate occupations, 10% tradespersons, shop or business owners or farmers, 24% executives or intellectual professions
Having a health‐related job	5%	5%	5%
Having a relative who has or had cancer	68%	67%	67%

## STUDY 1

The same correlations as in Fournier and Varet (2024) should be observed between pre‐exposure generic CBs, chemotherapy‐related CBs and treatment intentions (*H1* to *H3*). The following hypotheses consider the possibility that the health conspiracy article would make health‐related CBs more accessible among participants with a high (vs. low) level of pre‐exposure generic CBs exposure. Based on the previous considerations, exposure to a health conspiracy article (vs. no exposure) should be associated with more chemotherapy‐related CBs, even adjusting for pre‐exposure level of generic CBs (*H4*). Exposure to a health conspiracy article (vs. no exposure) should also be associated with a lower intention to use conventional medicine (*H5a*) and a higher intention to use non‐conventional medicine in complement (*H5b*), and in replacement (*H5c*), even when adjusting for pre‐exposure level of generic CBs. Finally, the relationships described in H5 should be mediated by chemotherapy‐related CBs (respectively *H6a*, *H6b* and *H6c* for each of the three treatment intentions).

### Methods of study 1

#### Participants and procedure

From October to November 2022, a total of 300 French participants with complete answer, were recruited via Prolific. Finally, 283 participants were retained for the analyses (see Figure 1 for details). After being automatically and randomly allocated to the Conspiracy or Control condition, participants were asked to complete the single‐item measure of generic CBs and the Beliefs about Medicine Questionnaire (BMQ). On the following page, a first distraction task was proposed to limit the risk of drawing links between the previous measures and the subsequent tasks and measures in the Conspiracy condition. This consisted of two matrices inspired by Raven's test of progressive matrices. To maintain comparability between conditions, the task was offered to participants in each condition.

Then, in the Conspiracy condition, participants were exposed to a fake newspaper article reporting the real Mediator scandal, a sanitary scandal that took place in France during the early 2010s. The article highlights the fact that different actors have conspired to promote financial interests over public health interests. The article was written by the authors from factual elements and events, based on three real press articles. For credibility purpose, the article was presented to the participants as coming from a French newspaper. A real scandal was chosen to ensure maximum credibility of the content presented. A scandal not related to cancer but to the wider field of health was chosen for two reasons. First, it should prevent participants from discovering the link between the experimental manipulation and the subsequent measures. Secondly, several observations from the literature suggest that experimental manipulation of CBs in one domain may influence CBs and health intentions in other domains. For example, Trella et al. (2024) found that exposure to real and fictitious CBs related to health or politics increases participants' conspiracy mentality. In Natoli and Marques (2021), exposure to antidepressant CBs reduced general trust in the health industry as well as various health‐seeking intentions. In Granados Samayoa et al. (2022), endorsement of health‐related CBs prospectively predicts greater endorsement of non‐health‐related CBs and a generalised conspiracy mindset. To ensure that participants read the article, a waiting time of 1 min 10 s was applied before being able to move on to the next page. In the Control condition, participants were not exposed to any text.

On the following page, a second distraction task, similar to the first, was proposed to participants in both conditions. To set a common representation of treatments before responding to the following questions, a short definition of chemotherapy and unconventional medicines was presented (see ‘Materials’). Participants then completed the chemotherapy‐related CBs scale. After being exposed to a projection task (i.e. vignette), they indicated their intention to use conventional and complementary and alternative medicines. They finally completed sociodemographic questions and the disturbance check item.

#### Measures

Additional details for the measurements presented below are available in ‘Study details’ and ‘Materials’ files.

##### Generic CBs

Generic CBs were measured with the single‐item CBs scale (SCIBS; Lantian et al., 2016). This measure consists of a statement: ‘I think that the official version of the events given by the authorities very often hides the truth’ for which participants are asked to assess its likelihood (from 1 = *Completely false* to 9 = *Completely true*).

##### General beliefs about medicines questionnaire

This measure (BMQ, Horne, 1999) includes two subscales that are Harm (e.g. treatments do more harm than good) and Overuse (e.g. doctors prescribe too many treatments). Details and associated secondary analyses are presented in the ‘Study details’ file.

##### Chemotherapy‐related CBs

The eight‐item scale from Fournier and Varet (2024) was used to measure chemotherapy‐related CBs (e.g. ‘The effectiveness of chemotherapy in treating cancer is disputed, but the pharmaceutical industry tries to hide this from the general public in order to make a profit.’). Items were rated on a 5‐point Likert scale (from 1 *Certainly not true* to 5 *Certainly true*) (*α* = .93).

##### Projection task and treatment intentions

Before measuring intentions, participants read a short fictional scenario in which they were asked to imagine themselves being diagnosed with cancer for which chemotherapy treatment is recommended. Intentions to use conventional, complementary and alternative medicine were measured with one item each for which participants were asked to indicate their accordance on a 5‐point Likert scale (from 1 *Totally disagree* to 5 *Totally agree*). Intention to use conventional medicine was negatively correlated with intention to use alternative medicine (*r* = −.62, *p* < .001) and not with intention to use complementary medicine (*r* = −.02, *p* = .69). Intentions to use alternative and complementary medicine were positively correlated (*r* = .20, *p* < .001).

##### Sociodemographic data

Sociodemographic data included gender, age, socio‐professional category, education, having a health‐related job, and personal and familial history of cancer.

#### Statistical analysis

Zero‐order Pearson correlation analyses were conducted to test the links between generic CBs, chemotherapy‐related CBs, intentions to use conventional medicine and CAM (*H1* to *H3*). Three mediation models were carried out to test the effect of the Conspiracy (vs. Control) condition on treatment intentions through adherence to chemotherapy‐related CBs (*H6*). Exploratory analyses were carried out in order to replicate Fournier and Varet (2024) results showing that the effects of generic CBs on treatment intentions are mediated by chemotherapy‐related CBs.

### Results of Study 1

#### Confirmatory analyses

Descriptive statistics and zero‐order Pearson correlations testing *H1* to *H3* are reported in the ‘Study details’ file. They replicate previous main findings from Fournier and Varet (2024).

**FIGURE 2:**
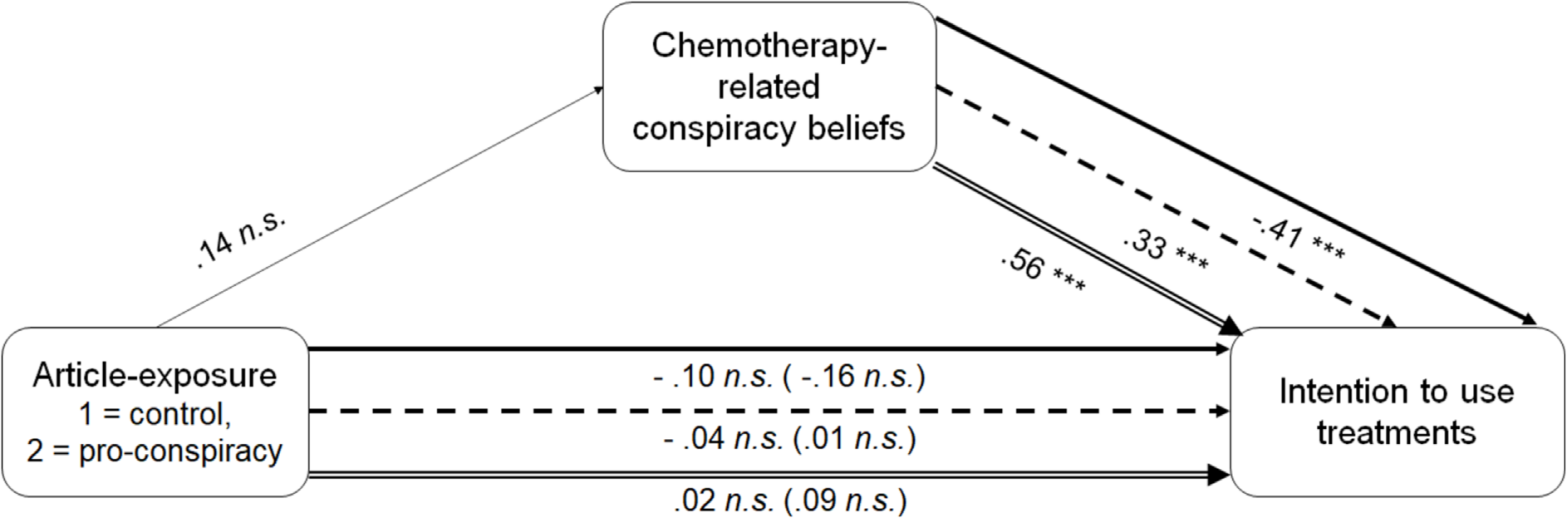
Mediation models of the effect of the condition on intentions to use treatments, through chemotherapy‐related CBs, controlling for pre‐exposure CBs. The results of the three moderated‐mediation models are presented on the same figure for practical reasons. Paths with a continuous arrow (up) refer to the model for the intention to use conventional medicine. Paths with a dashed arrow (middle) refer to the model for the intention to use complementary medicine. Paths with a double‐edged arrow (low) refer to the model for the intention to use alternative medicine. Numbers from the condition to other variables represent Cohen's *d* effect sizes. Numbers from chemotherapy‐related CBs to intention to use treatment represent standardised beta coefficients. Number of bootstrap samples for percentile bootstrap confidence intervals = 5000. *n.s*. = Non‐significant: *p* > .10, ***: *p* < .001.

Three mediation models were carried out with the condition as independent variable (1 = Control condition; 2 = Conspiracy condition), chemotherapy‐related CBs as the mediator and intention to use conventional medicine, complementary medicine or alternative medicine as the outcome (Model 4 in PROCESS macro). The results indicated no significant effect of the condition on chemotherapy‐related CBs (Cohen's *d* = .10, *p* = .387). Total effects of the condition on each of the three treatment intentions were not significant (all *p*s ≥ .2469). The indirect effects of the condition on each of the three treatment intentions, through chemotherapy‐related CBs, were not significant (for conventional medicine: Cohen's *d* = −.05, 95% CI [−.15; .06]; for complementary medicine: Cohen's *d* = .03, 95% CI [−.04; .12]; for alternative medicine: Cohen's *d* = .06, 95% CI [−.08; .20]). These results were unchanged when pre‐exposure generic CBs were adjusted for (see Figure 2). Thus, *H6* was rejected.

#### Exploratory analyses

Mediations analyses replicated one of the main results from Fournier and Varet (2024) where the effects of generic CBs on each of the treatment intentions are mediated by chemotherapy‐related CBs (for detailed results see ‘Study details’ file).

### Discussion of Study 1

As in Fournier and Varet (2024), generic CBs and chemotherapy‐related CBs are highly correlated with each other and linked with less intention to use conventional medicine and more intention to use CAM, especially alternative medicine, with medium to large effect sizes. The effect of generic CBs on each treatment intention was found to be mediated by chemotherapy‐related CBs. In addition, this study was conducted as an attempt to experimentally manipulate cancer‐related CBs by presenting a real scandal (vs. not presenting one) about a conspiracy related to health but not to cancer. Contrary to what was expected, this was not found to be effective in manipulating adherence to cancer‐related CBs. Several explanations can be envisaged. The experimental manipulation may have been effective in increasing CBs specific to the presented scandal (e.g. towards the Mediator drug), without generalising more widely to other health field such as cancer treatments. Otherwise, the experimental manipulation may have not been effective in increasing any CBs because the article was not considered credible by the participants. These speculations cannot be verified as CBs related to the Mediator scandal and perceived credibility of the article were not measured. Thus, Study 2 was designed to manipulate cancer‐related CBs using a different material.

## STUDY 2

Study 2 aims to test the same hypotheses as in Study 1, with the exception that generic CB will not be adjusted for in the analyses, as they will be measured after rather than before the experimental manipulation. In addition, exposure to a pro‐conspiracy article will be compared to exposure to an anti‐conspiracy article rather than no exposure.

### Methods of Study 2

#### Participants and procedure

In January 2023, 258 participants fluent in French and from France, Belgium or Luxembourg were recruited via Prolific. Finally, 242 participants were retained for the analyses (see Figure 1 for details). They were automatically and randomly allocated to the Pro‐conspiracy or Anti‐conspiracy condition. To avoid possible interference, no measurement was offered before the experimental manipulation. In the Pro‐conspiracy condition, participants were exposed to a fake newspaper article supporting the existence of a health conspiracy (i.e. contesting the effectiveness of chemotherapy to treat cancer and pointing to the role of the pharmaceutical industry in falsifying evidence and serving its financial interests). In the Anti‐conspiracy condition, participants were exposed to a fake newspaper article arguing against the existence of a health conspiracy (i.e. affirming the effectiveness of chemotherapy to treat cancer and the absence of a conspiracy by the pharmaceutical industry). For credibility purposes, articles were presented as short excerpts from a French newspaper. The characteristics of the articles (i.e. structure, length, type of argument and vocabulary) were as close as possible to those of Natoli and Marques (2021), based on those on Jolley and Douglas (2014). As in Study 1, a waiting time of 1 min 10 s was applied before being able to move on to the next pages where a short definition of chemotherapy and unconventional medicines was presented. Participants were then asked to complete the following measures.

#### Measures

The measures are the same as in Study 1 and presented in the following order to the participants: chemotherapy‐related CBs (*α* = .94), treatment intentions (assessed after reading the vignette), BMQ, generic CBs and sociodemographic data. At the end, the perceived credibility of the newspaper article was measured with the unidimensional three‐item scale from Appelman and Sundar (2016) (accurate/authentic/credible; from 1 *Describes very poorly* to 7 *Describes very well*; *α*_pro‐conspiracy_ = .89; *α*_anti‐conspiracy_ = .86). The items and instructions were translated and adapted from English into French by the authors.

#### Statistical analysis

As in Study 1, *H1* to *H3* were tested with zero‐order Pearson correlations, while *H4* to *H6* were tested with mediation analyses. Exploratory analyses include the reiteration of the mediation analyses adding the perceived credibility of the article as a moderator of the effect of the experimental manipulation on chemotherapy‐related CBs (i.e. moderated mediation).

### Results of Study 2

#### Confirmatory analyses

Similar correlations patterns to those found in Study 1 were found for generic CBs, chemotherapy‐related CBs and treatment intentions, corroborating *H1* to *H3* (see ‘Study details’ file). Three mediation models were carried out with the condition as the independent variable (1 = Anti‐conspiracy condition; 2 = Pro‐conspiracy condition), chemotherapy‐related CBs as the mediator and the intention to use conventional medicine, complementary medicine or alternative medicine as the outcome. The results indicated that the Pro‐conspiracy (vs. Anti‐conspiracy) condition was associated with more chemotherapy‐related CBs (Cohen's *d* = .66, *p* < .001), supporting *H4*. The Pro‐conspiracy (vs. Anti‐conspiracy) condition had no significant total effect on intentions to use conventional medicine (*p* = .1452) or complementary medicine (*p* = .0776) but did have an effect on intention to use alternative medicine (Cohen's *d* = 0.32, *p* = .0119), partially supporting *H5*. The condition had no significant direct effect on each of the treatment intentions (*p*_conventional medicine_ = .0633, *p*_complementary medicine_ = .7622, *p*_alternative medicine_ = .2624). However, the condition had a significant indirect effect on each of the three treatment intentions, through chemotherapy‐related CBs, with small effect sizes (for conventional medicine: Cohen's *d*_indirect effect_ = −.40, 95% CI [−.57, −.24]; for complementary medicine, Cohen's *d*_indirect effect_ = .27, 95% CI [.15, .40]; for alternative medicine, Cohen's *d*_indirect effect_ = .44, 95% CI [.28, .61]), supporting *H6*.

#### Exploratory analyses

Student *t*‐tests revealed that the Pro‐ and Anti‐conspiracy articles are respectively associated with an average perceived credibility below and above the central value of the scale, with a significant difference between the two articles (see ‘Study details’ file for details).

The perceived credibility of a source of persuasion is likely to condition its effects on attitudes. Indeed, a source perceived as not credible may have a weaker persuasive effect than a source perceived as highly credible, or even the opposite of the desired effect (e.g. backfire effect) (Pornpitakpan, 2004). For this reason, the three mediation models presented in the confirmatory analyses were specified adding the perceived credibility of the article as a moderator of the effect of condition on chemotherapy‐related CBs (Model 7 in PROCESS macro).

**FIGURE 3:**
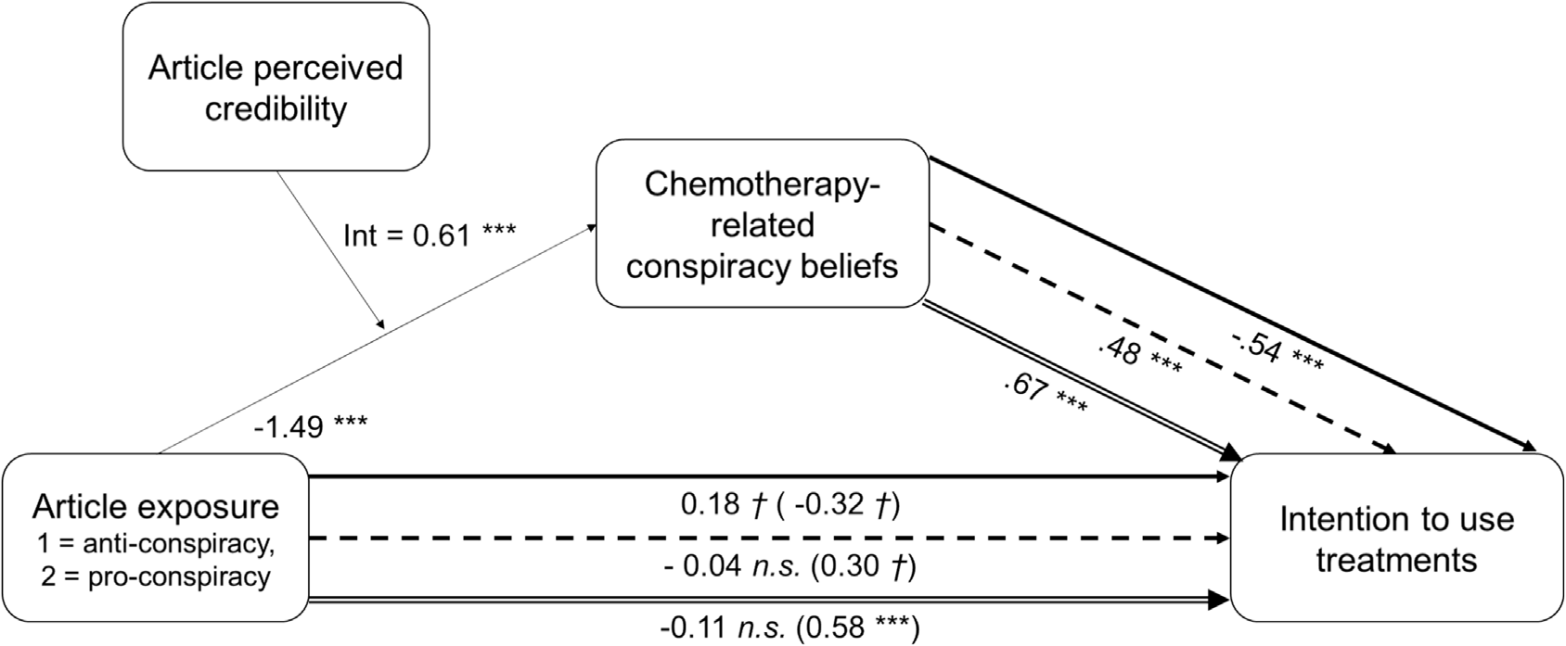
Moderated‐mediation models of the effect of the condition on intentions to use treatments, through chemotherapy‐related CBs. The path from the condition to chemotherapy‐related CBs is moderated by the perceived credibility of the article. The results of the three moderated‐mediation models are presented on the same figure for practical reasons. Paths with a continuous arrow (up) refer to the model for the intention to use conventional medicine. Paths with a dashed arrow (middle) refer to the model for the intention to use complementary medicine. Paths with a double‐edged arrow (low) refer to the model for the intention to use alternative medicine. Numbers from the condition to other variables represent Cohen's *d* effect sizes. Numbers from chemotherapy‐related CBs to intention to use treatment represent standardised beta coefficients. Number of bootstrap samples for percentile bootstrap confidence intervals = 5000. *n.s*. = Non‐significant: *p*> .10, *†*: *p* > .05, ***: *p* < .001.

**FIGURE 4:**
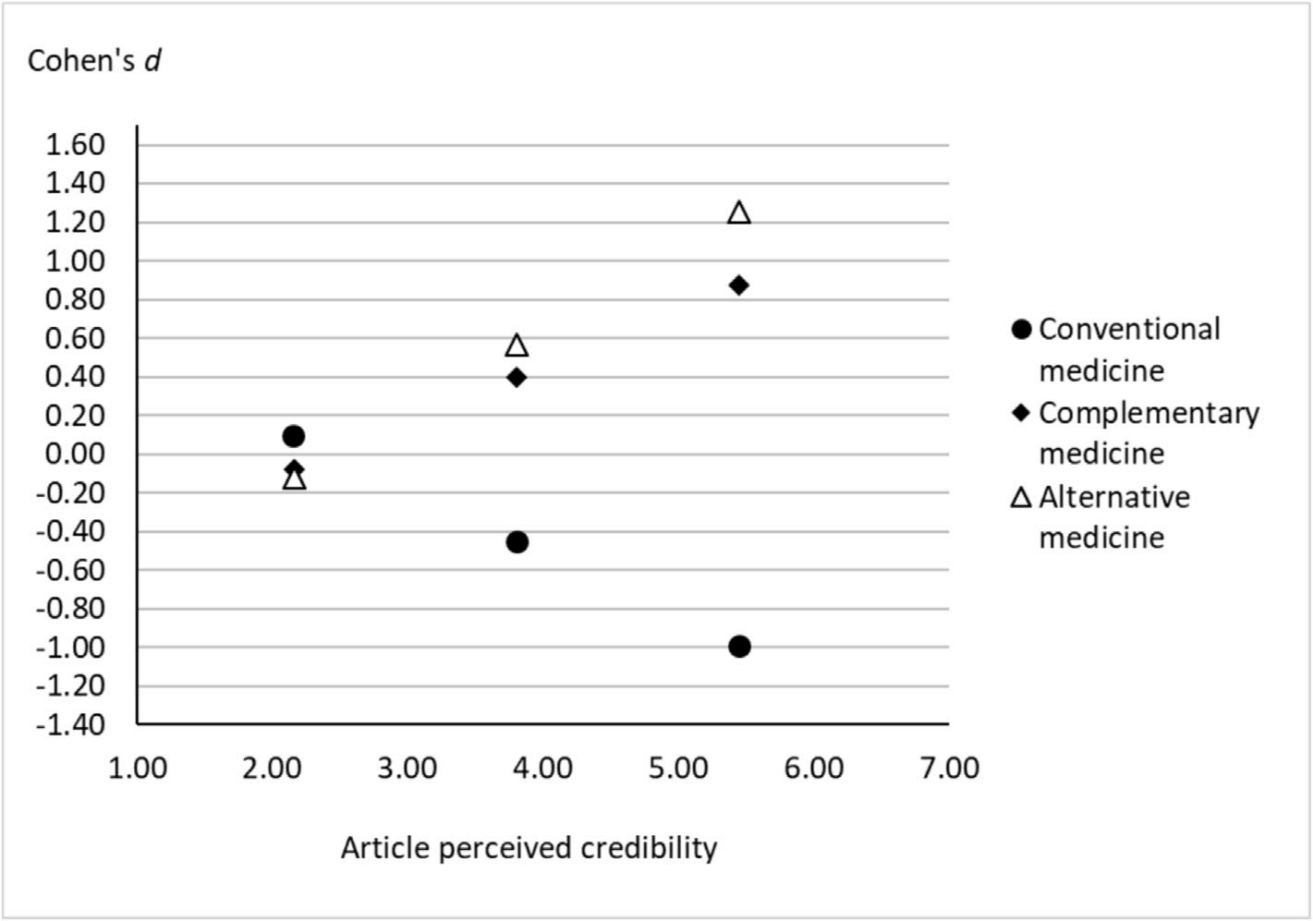
Conditional indirect effect of the pro‐conspiracy (vs. anti‐conspiracy) condition on intention to use treatment through chemotherapy‐related conspiracy beliefs, according to the perceived credibility of the article. Perceived credibility of the article is considered at the −1 S.D. from the mean value (2.16), at the mean value (3.81) and at the +1 S.D. from the mean value (5.45).

As shown in Figure 3, the results indicate a significant Condition*Credibility interaction (unstandardised β = .61, *p* < .001, *R* ^2^ change = .22) and a significant index of moderated mediation for each of the three treatment intentions (for conventional medicine, *index* = −.33; 95% CI [−.44, −.23]; for complementary medicine, *index* = .29; 95% CI [.20, .40]; for alternative medicine, *index* = .42; 95% CI [.30, .54]). As shown in Figure 4, conditional indirect effects (through chemotherapy‐related CBs) indicate that, at a high level of article credibility (i.e. +1 *SD* from the mean = 5.45), the Pro‐conspiracy (vs. Anti‐conspiracy) condition was associated with less intention to use conventional medicine, with a large effect size (Cohen's *d* = −0.99, 95% CI [−1.29, −0.71]), while no difference is observed at a low level of article credibility (i.e. −1 *SD* from the mean = 2.16) (Cohen's *d* = 0.09, 95% CI [−0.07, 0.27]). At a high level of article credibility, the Pro‐conspiracy (vs. Anti‐conspiracy) condition is associated with more intention to use complementary medicine, with a large effect size (Cohen's *d* = 0.88, 95% CI [0.58, 1.19]), while no difference is observed at a low level of article credibility (Cohen's *d* = −0.08, 95% CI [−0.23, 0.06]). Similarly, at a high level of article credibility, the Pro‐conspiracy (vs. Anti‐conspiracy) condition is associated with more intention to use alternative medicine, with a large effect size, (Cohen's *d* = 1.25, 95% CI [0.95, 1.58]), while no difference is observed at a low level of article credibility (Cohen's *d* = −0.12, 95% CI [−0.34, 0.09]). A posteriori power analyses revealed an insufficient achieved power to detect indirect effects of article exposure on treatment intentions (all powers ≤ .40), but sufficient achieved power to detect conditional indirect effects (all powers = 1.00) and the moderating effect of credibility on the path from condition to chemotherapy‐related CBs (all powers = 1.00) (see ‘Study details’ file).

### Discussion of Study 2

Exposure to a pro‐conspiracy (vs. anti‐conspiracy) article about cancer treatments appears to be effective in experimentally manipulating chemotherapy‐related CBs. In addition, through increasing chemotherapy‐related CBs, exposure to the Pro‐conspiracy (vs. Anti‐conspiracy) article indirectly influences treatment intentions. Importantly, the exploratory analyses indicate that these indirect effects are considerably stronger among participants perceiving the article as highly credible, while being non‐existent or reversed among participants perceiving the article as weakly credible. The moderating effect of the perceived credibility on persuasion is already well known in the literature (Pornpitakpan, 2004). However, this effect has not been empirically investigated in the specific context of cancer misinformation (Swire‐Thompson & Johnson, 2024). To counter the effects of a cancer misinformation content, weakening its perceived credibility could be effective. Given the credibility was not experimentally manipulated, it is possible that participants who associated a high (vs. low) credibility to the Anti‐conspiracy article and a low (vs. high) credibility to the Pro‐conspiracy article previously endorsed a low (vs. high) level of CBs. Given CBs were measured after the article exposure, this hypothesis cannot be rejected. In addition, the pro‐conspiracy article was rated as less credible than the anti‐conspiracy article. Thus, Study 3 was designed to experimentally manipulate the article credibility, in addition to its exposure.

## STUDY 3

Study 3 has two aims. First, it aims to replicate the effect of exposure to a conspiracy content about chemotherapy and the effect of its perceived credibility on treatment intentions using a fully experimental design. Secondly, it aims to test the effectiveness of a brief intervention to prevent the effect of conspiracy content exposure, which is allowed by the experimental manipulation of the perceived credibility of the content.

The intervention consists of presenting, before the exposition to misinformation, a short message combining two kinds of levers used in several interventions against misinformation: a *warning prompt* and an *accuracy prompt* (Kozyreva et al., 2024). Warning prompts consist in explicitly alerting about the risk of being exposed to erroneous information, while accuracy prompts aim to draw attention to the concept of accuracy (Kozyreva et al., 2024). Warning *prompts* should be distinguished from warning *labels*. Warning labels consists of an alert directed at a specific content presented at the same time (Martel & Rand, 2023). Warning prompts are a general warning about all contents, presented before exposure. Although warning prompts and accuracy prompts can theoretically be distinguished, exposure to a warning prompt is often included in interventions labelled with ‘accuracy prompt’ (e.g. Lin et al., 2024). The interest of accuracy prompts to reduce misinformation sharing for online contents was found to be robust and generalizable in health‐related and non‐health‐related settings (Pennycook & Rand, 2022). However, few studies have investigated the effects of online accuracy prompts on offline behaviours or behavioural intentions. There seem to be even fewer studies in the field of health, and none in the field of oncology (Swire‐Thompson & Johnson, 2024).

It is expected that treatment intentions show the same correlations with chemotherapy‐related CBs as in Study 1 and Study 2 (*H1a* to *H1c*). Treatment intentions should also be correlated in the same direction with perceived credibility (*H2a* to *H2c*). The Prevention condition (vs. Conspiracy condition) should be associated with a lower perceived credibility of the article (*H3a*), lower chemotherapy‐related CBs (*H3b*), higher intention to use conventional medicine (*H3c*), and lower intentions to use non‐conventional medicine in complement (*H3d*), and in replacement (*H3e*). Chemotherapy‐related CBs should mediate the effect of perceived credibility of the article on treatment intentions (*H4a* to *H4c*). Finally, perceived credibility of the article (Mediator 1) and chemotherapy‐related CBs (Mediator 2) should serially mediate the difference between the Prevention and the Conspiracy conditions on treatment intentions (H5a to H5c).

### Methods of Study 3

#### Participants and procedure

From October to November 2023, a total of 462 French participants with complete answers were recruited via the online recruitment platform Foule Factory. Finally, 386 participants were retained for the analyses (see Figure 1 for details). They were automatically and randomly allocated to the Control condition, Conspiracy condition or Prevention condition. For all three conditions, participants were then presented with a page including a message stating that the questionnaire is loading and that the button to go to the next page will appear in a few seconds (the page was programmed to make this message flash and disappear after 20 s, when the ‘next’ button appears). In the Prevention condition only, this page also included a short message combining a *warning prompt* and an *accuracy prompt*. The message appeared on the page in the same style as the survey platform tips and help messages. This message was ‘Faced with the proliferation of false information on the internet and social networks, we would like to draw your attention to the need to be vigilant about the sources, arguments, and media of the information you are confronted with. It is vital to exercise this vigilance daily to guard against manipulation and develop a critical mind.’ In the Control condition and Conspiracy condition, no message was presented.

On the following pages, in each condition, the same distraction task as in Study 1 was proposed to prevent participants in the Prevention condition from making connections between the prompt message and the subsequent measures. Participants in the Prevention and Conspiracy conditions were exposed to the same fake newspaper article as in Study 2. In the Control condition, participants were not exposed to any text. For all three conditions, participants were then asked to complete the following measures.

#### Measures

The following measures are the same as in Study 2 and presented in this order to the participants: chemotherapy‐related CBs (*α* = .97), treatment intentions (assessed after reading the vignette), BMQ, generic CBs, sociodemographic data, credibility of the newspaper article (*α*_conspiracy_ = .89; *α*_prevention_ = .89). Likert scales were seven points for all psychological measures, except for treatment intentions, which were rated with sliders from 0 *Totally disagree* to 100 *Totally agree*. An additional single item using a bipolar scale opposing intention to use CAM and conventional medicine was also used. Results including this item are presented in the ‘Study details’ file, as the conclusions of the analyses remained unchanged compared to those with the two distinct intentions to use alterative and conventional medicine, and as this item was not pre‐registered.

#### Statistical analysis

As in Study 1 and Study 2, the links between chemotherapy‐related CBs and treatment intentions (*H1*, *H2*) were tested with zero‐order Pearson correlations. Three serial mediation models were used to test the successive mediator role of perceived credibility of the article and chemotherapy‐related CBs in explaining the effect of the condition on treatment intentions (*H3*, *H4*, *H5*). Exploratory analyses examined possible differences in chemotherapy‐related CBs and intentions to use treatments between Control conditions and, respectively, Conspiracy and Prevention conditions.

### Results of Study 3

#### Confirmatory analyses

Descriptive statistics and zero‐order Pearson correlations testing *H1* to *H2* are reported in the ‘Study details’ file.

**FIGURE 5:**
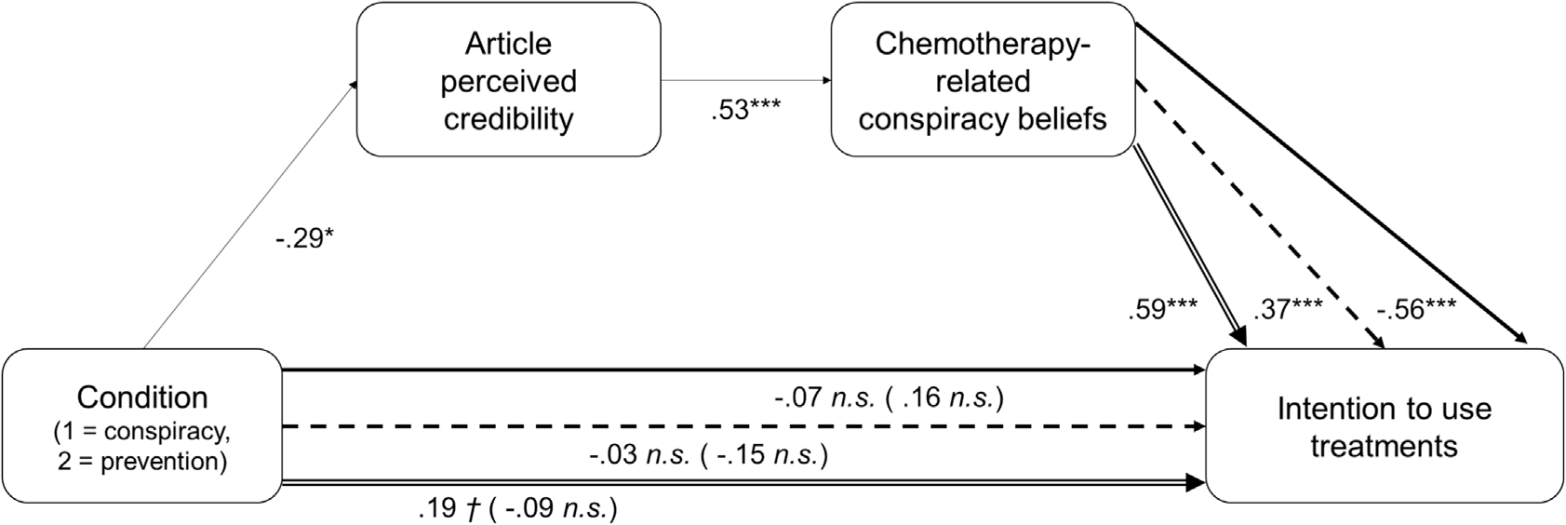
Serial mediation models of the effect of the condition on intentions to use treatments, through article perceived credibility and chemotherapy‐related conspiracy beliefs. The results of the three serial mediation models are presented on the same figure for practical reasons. Paths with a continuous arrow (up) refer to the model for the intention to use conventional medicine. Paths with a dashed arrow (middle) refer to the model for the intention to use complementary medicine. Paths with a double‐edged arrow (low) refer to the model for the intention to use alternative medicine. Numbers from condition to other variables represent Cohen's *d* effect sizes. Numbers from article perceived credibility, and chemotherapy‐related conspiracy beliefs, to intention to use treatment represent standardised beta coefficients. Number of bootstrap samples for percentile bootstrap confidence intervals = 5000. *n.s*. = Non‐significant: *p* > .10, *†*: *p* > .05, ***: *p* < .001.

Three serial mediation models (Model 6 in PROCESS macro) were carried out with the condition (1 = Conspiracy condition; 2 = Prevention condition) as the independent variable, credibility of the article as the first mediator, chemotherapy‐related CBs as the second mediator and intention to use conventional medicine, complementary medicine or alternative medicine as the outcomes. The results (see Figure 5) indicate that the article is perceived as less credible in the Prevention condition than in the Conspiracy condition, with a small effect size (Cohen's *d* = −.29, *p* = .022), supporting *H3a*. When perceived credibility of the article is accounted for, participants reported less chemotherapy‐related CBs in Prevention than in Conspiracy condition, with a small effect size (Cohen's *d* = −.24, *p* = .023), supporting *H3b*. Article exposure had no significant total effect on the three treatment intentions (*p*_conventional medicine_ = .1969, *p*_complementary medicine_ = .2307, *p*_alternative medicine_ = .499), not supporting *H3c*, *H3d*, *H3e*, nor significant direct effect on the three treatment intentions (*p*_conventional medicine_ = .5422, *p*_complementary medicine_ = .7890, *p*_alternative medicine_ = .053).

Regarding intention to use conventional medicine, the indirect effect of the condition through credibility was not significant (Cohen's *d* = 0.01, 95% CI [−0.03, 0.05]). The indirect effect through chemotherapy‐related CBs was significant, with a small effect size (Cohen's *d* = 0.14, 95% CI [0.02, 0.26]), as well as the indirect effect through both serial mediators (Cohen's *d* = 0.09, 95% CI [0.01, 0.17]) and the aggregated indirect effects (Cohen's *d* = 0.23, 95% CI [0.09, 0.38]), with a higher intention to use conventional medicine in the Prevention condition than in the Conspiracy condition. Regarding intention to use complementary medicine, the indirect effect of the condition through credibility was not significant (Cohen's *d* = 0.03, 95% CI [−0.02, 0.09]). The indirect effect through chemotherapy‐related CBs was significant, with a small effect size (Cohen's *d* = −0.09, 95% CI [−0.19, −0.01]), as well as the indirect effect through both serial mediators (Cohen's *d* = −0.06, 95% CI [−0.12, −0.01]) and the aggregated indirect effects (Cohen's *d* = −0.12, 95% CI [−0.22, −0.03]), with a lower intention to use complementary medicine in the Prevention condition than in the Conspiracy condition. Regarding intention to use alternative medicine, all the indirect effects of the condition were significant, with small effect sizes, with a lower intention to use alternative medicine in Prevention condition than in Conspiracy condition (for the indirect effect through credibility: Cohen's *d* = −0.04, 95% CI [−0.11, −0.0007]; for the indirect effect through chemotherapy‐related CBs: Cohen's *d* = −0.14, 95% CI [−0.28, −0.02]; for the indirect effect through both serial mediators: Cohen's *d* = −0.09, 95% CI [−0.17, −0.01]; for the aggregated indirect effects: Cohen's *d* = −0.27, 95% CI [−0.45, −0.11]). *H4* and *H5* were therefore supported.

A posteriori power analyses revealed an insufficient achieved power to detect indirect effects of the condition on treatment intentions through credibility only (all powers ≤ .69), but a sufficient achieved power to detect indirect effects through chemotherapy‐related CBs only (all powers ≥99) and through both credibility and chemotherapy‐related CBs (all powers = 1.00) (see ‘Study details’).

#### Exploratory analyses

Two sets of three mediation models were tested with the condition as independent variable. In the first set, the Control condition (=1) was compared to the Conspiracy condition (=2). In the second set, the Control condition (=1) was compared to the Prevention condition (=2). In all models, chemotherapy‐related CBs were the mediator, and intention to use conventional medicine, complementary medicine or alternative medicine was the outcome (Model 4 in PROCESS macro). Fort the first set, the results indicated that the Conspiracy condition (vs. Control condition) was associated with more chemotherapy‐related CBs (Cohen's *d* = .52, *p* < .001). Regarding total effects, the Conspiracy (vs. Control condition) was associated with less intention to use conventional medicine (Cohen's *d* = −.35, *p* = .0042) but not with significant differences for intentions to use complementary medicine (Cohen's *d* = .13, *p* = .2842) nor alternative medicine (Cohen's *d* = .21, *p* = .0930). The condition had no significant direct effect on each of the three treatment intentions (*p*_conventional medicine_ = .5270, *p*_complementary medicine_ = .8262, *p*_alternative medicine_ = .2070). However, the condition had a significant indirect effect on each of the three treatment intentions, through chemotherapy‐related CBs, with small effect sizes (for conventional medicine: Cohen's *d*_indirect effect_ = −.28, 95% CI [−.43, −.15]; for complementary medicine, Cohen's *d*_indirect effect_ = .16, 95% CI [.07, .26]; for alternative medicine, Cohen's *d*_indirect effect_ = .33, 95% CI [.18, .50]). For the second set, the results indicated that, compared to the Control condition, the Prevention condition was associated with no significant difference on chemotherapy‐related CBs (Cohen's *d* = .06, *p* = .3809). Regarding total effects, the condition was associated with no significant differences on each of the three treatment intentions (*p*_conventional medicine_ = .1534, *p*_complementary medicine_ = .9039, *p*_alternative medicine_ = .3513). The condition had no significant direct effect on each of the three treatment intentions (*p*_conventional medicine_ = .2589, *p*_complementary medicine_ = .6788, *p*_alternative medicine_ = .6235). Finally, the condition had no significant indirect effect on each of the three treatment intentions, through chemotherapy‐related CBs (for conventional medicine: Cohen's *d*_indirect effect_ = −.03, 95% CI [−.10, .04]; for complementary medicine, Cohen's *d*_indirect effect_ = .02, 95% CI [−.02, .06]; for alternative medicine, Cohen's *d*_indirect effect_ = .03, 95% CI [−.04, .12]).

### Discussion of Study 3

Compared to the Conspiracy condition, the mediation analyses showed no significant total effect of the Prevention condition on the intention to use treatments. When the perceived credibility is considered as the only mediating variable, the results show an indirect effect of the Prevention (vs. Conspiracy) condition on the intention to use alternative medicine, but not for complementary and conventional medicines. Importantly, for the three treatment intentions, a significant indirect effect through chemotherapy‐related CBs only and through credibility and then chemotherapy‐related CBs consecutively was found. Exposure to the warning and accuracy prompt (vs. no prompt), before exposure to the misinformation, was indirectly associated with more intention to use conventional medicine and less intention to use complementary and alternative medicine. Exploratory analyses found no differences between the Control and Prevention conditions on chemotherapy‐related CBs and the three treatment intentions. However, the Conspiracy condition was associated with more chemotherapy‐related CBs and a lower intention to use conventional medicine than the Control condition. Compared with a baseline situation, this suggests that the Prevention condition would be effective in preventing some of the harmful effects observed in the Conspiracy condition.

By experimentally manipulating both exposure to CBs and their perceived credibility, these results corroborate the existence of detrimental effects of chemotherapy‐related CBs on treatment intentions, as suggested by Fournier and Varet (2024). Fortunately, the results also suggest that this detrimental effect could be prevented by exposure to a combination of warning and accuracy prompts, prior to exposure to a misinformation conveying CBs. The effectiveness of previous interventions based on accuracy prompts to counter online health‐related misinformation has mostly been established for online behaviours (e.g. social network sharing) (Pennycook & Rand, 2022; Swire‐Thompson & Johnson, 2024). Interestingly, the results of the present study corroborate their effectiveness in preventing misinformation effects on offline and health‐related behavioural intentions, particularly in oncology.

## GENERAL DISCUSSION

Using a cancer‐free population and a projection task, the three studies of the present research aimed to experimentally investigate the effect of endorsing cancer‐related CBs, conveyed through online misinformation, on the intentions to use conventional medicine and CAM. Study 1 was conducted to experimentally manipulate cancer‐related CBs by presenting a real scandal related to health but not cancer (vs. not presenting one) but failed to do so. This could be due to a non‐generalisation of activated CBs to cancer treatments, or failure to activate any CBs due to lack of credibility. It could also be that the article only influenced chemotherapy‐related CBs in participants who perceived it as highly credible, but this credibility was not measured. Study 2 and Study 3 therefore proposed to manipulate cancer‐related CBs with exposure to an online misinformation article conveying CBs about cancer treatments. Study 2 showed that exposure to this article, compared to an anti‐conspiracy article, indirectly affects treatment intentions by increasing chemotherapy‐related CBs. Participants exposed to the conspiracy content reported less intention to conventional medicine and higher intention to use CAM when asked to put themselves in the shoes of a cancer patient. Importantly, exploratory analyses indicated that these effects were considerably stronger among participants perceiving the article as highly credible, while being non‐existent or reversed among participants perceiving the article as weakly credible. However, credibility was not a manipulated variable, and the pro‐conspiracy article was rated as low credibility and the anti‐conspiracy article as high credibility. Study 3 was therefore carried out to conceptually replicate these results with an experimental manipulation of the perceived credibility and to test a brief intervention to prevent the harmful effect of misinformation exposure by decreasing its perceived credibility. The brief intervention consisted in presenting to participants a warning and accuracy prompt before being exposed to the online misinformation. Compared to a Control condition with no intervention nor conspiracy content exposure, conspiracy content exposure only was associated with more chemotherapy‐related CBs and lower intention to use conventional medicine. Compared to conspiracy content exposure only, implementing the warning and accuracy prompt was directly associated with less chemotherapy‐related CBs and indirectly associated with more intention to use conventional medicine and less intention to use CAM.

### Misinformation, CBs and health behaviours

Taken together, the results presented above corroborate the existence of an effect of cancer‐related CBs, when conveyed by online misinformation perceived as credible, on intention to use CAM and to not use conventional medicine. This finding is worrying for public health, as people with cancer or at risk of developing it can be frequently exposed to online cancer misinformation (Swire‐Thompson & Johnson, 2024) and as refusal of or poor adherence to conventional oncology treatments impairs survival and quality of life, including among patients with curable cancer (Jacobs et al., 2019; Johnson et al., 2018a,b). In addition, supplementary analyses in Studies 1 and 2 indicate that exposure to misinformation conveying cancer CBs also increased generic CBs and negative general beliefs about conventional medicine (see ‘Study details’ file). This suggests that cancer misinformation may also have detrimental effects on health behaviours that are not solely cancer‐related, making it a broader health issue.

### Dealing with CBs in public health and oncology

Study 3 corroborates the effectiveness of a brief intervention, based on a warning and accuracy prompt, to prevent some of the detrimental effects of cancer misinformation, by reducing its perceived credibility and chemotherapy‐related CBs. This brief intervention has the advantage of being simple and rapid and could be of particular interest if coupled with an automatic detection system for medical misinformation contents implemented in social media applications, websites or browsers (e.g. Zhu et al., 2024). Other types of interventions aimed at counteract cancer‐related misinformation and conspiracy theories by weakening their credibility could also be developed and tested in oncology. For example, debunking, prebunking and media literacy interventions were found to be effective to improve misinformation credibility assessment or reduce their endorsement, although effect sizes are often small to medium, and long‐term effectiveness remains little studied (Heley et al., 2024; Lu et al., 2024; Oliveira et al., 2024).

Fighting misinformation can also involve paying particular attention to the credibility that people attribute to real information, in particular those that attempt to debunk misinformation (Acerbi et al., 2022). Indeed, the results from Study 2 suggest that if an intervention arguing against cancer‐related conspiracy theories is perceived as lacking credibility, it may be ineffective in reducing related beliefs and intention to use CAM and increasing use of conventional medicine. To avoid wasting resources, it therefore seems important that health communication campaigns in oncology are systematically pre‐tested before being rolled out on a larger scale, as this does not always seem to be the case (e.g. in the case of HIV prevention, see Lacroix et al., 2014). Interventions aiming at fostering confidence in reliable information have received less attention and remain to be developed and tested (Acerbi et al., 2022). Interestingly, other levers for action could be envisaged via the training and support of oncology healthcare professionals. For example, improving patient‐centred and empathetic communication could reduce the effect of medical mistrust on patient's decision making (Cuevas et al., 2019) and prevent medical CBs (Marques et al., 2022).

### Limitations

This study makes a significant contribution to the literature on the consequences of misinformation and CBs in oncology. However, several limitations should be noted. Moderated‐mediation and double serial mediation models are based on a number of assumptions, which have not all been investigated here and which therefore limit their ability to validate causal claims (Rohrer et al., 2022). In the three studies, chemotherapy‐related CBs were conceptualised as a mediator rather than as a manipulation check of article exposure. Consequently, it would have been preferable to introduce a dedicated manipulation check, such as a recall, recognition or comprehension test. Study 2 was insufficiently powered to detect the direct effects of exposure to the pro‐conspiracy (vs. anti‐conspiracy) article on treatment intentions. Similarly, Study 3 was underpowered to detect the indirect effects of the condition (conspiracy vs. prevention) on treatment intentions through credibility only. However, both studies were sufficiently powered to detect the indirect effect of the experiment manipulation through chemotherapy‐related CBs, which was more central to their objective. As in Fournier and Varet (2024), the samples were composed of cancer‐free participants from the general population exposed to a fictitious situation. This methodological setting was mainly justified by avoiding exposing patients with cancer to a potentially harmful situation (Bradbury‐Jones et al., 2014). However, fictitious situations may produce different results from more ecological situations (Atzmüller & Steiner, 2010). Having cancer could have a significant impact on factors that affect susceptibility to misinformation and CBs, such as emotions, health literacy and beliefs about treatments and health professionals. Another limitation of the present research is that conventional medicine was only considered through chemotherapy, which may be subject to specific perceptions compared to other treatments, on the part of the general population and patients.

## CONCLUSION AND PERSPECTIVES

This research highlights the harm of misinformation and CBs on health behaviours, especially in oncology. Future studies should investigate the effects of misinformation and CBs on adherence to several types of conventional treatments other than chemotherapy in patients with cancer. This paper highlights that anti‐misinformation communication may be ineffective, underscoring the need for pre‐testing before implementation. Given the importance of cancer for public health, there is an urgent need to carry out studies not only on patients in order to develop specific interventions but also on the general population in order to develop preventive measures.

## CONFLICT OF INTEREST STATEMENT

The authors declare that they have no known competing financial interests or personal relationships that could have appeared to influence the work reported in this paper.

## ETHICS STATEMENT

All studies received the agreement of the Research Ethics Board of the University of Lille (France, reference number 2022‐603‐S105). They were conducted in accordance with the 1964 Helsinki Declaration and its later amendments, the ethical principles of the French Code of Ethics for Psychologists and the 2016 APA Ethical Principles of Psychologists and Code of Conduct.

## Data Availability

The data that support the findings of this study are openly available in OSF at https://doi.org/10.17605/OSF.IO/2EDT9.

## References

[aphw12615-bib-0001] Acerbi, A. , Altay, S. , & Mercier, H. (2022). Research note: Fighting misinformation or fighting for information? Harvard Kennedy School Misinformation Review. 10.37016/mr-2020-87

[aphw12615-bib-0003] Appelman, A. , & Sundar, S. S. (2016). Measuring message credibility: Construction and validation of an exclusive scale. Journalism and Mass Communication Quarterly, 93(1), 59–79. 10.1177/1077699015606057

[aphw12615-bib-0004] Atzmüller, C. , & Steiner, P. M. (2010). Experimental vignette studies in survey research. Methodology, 6(3), 128–138. 10.1027/1614-2241/a000014

[aphw12615-bib-0005] Ben Kridis, W. , Mnif, A. , Khmiri, S. , Toumi, N. , & Khanfir, A. (2021). Self‐medication with herbal medicine and breast cancer survival: A prospective monocentric study. Journal of Cancer Research and Clinical Oncology, 147(11), 3401–3407. 10.1007/s00432-021-03600-y 33748880 PMC11802091

[aphw12615-bib-0006] Bradbury‐Jones, C. , Taylor, J. , & Herber, O. R. (2014). Vignette development and administration: A framework for protecting research participants. International Journal of Social Research Methodology, 17(4), 427–440. 10.1080/13645579.2012.750833

[aphw12615-bib-0007] Braun, L. A. , Zomorodbakhsch, B. , Keinki, C. , & Huebner, J. (2019). Information needs, communication and usage of social media by cancer patients and their relatives. Journal of Cancer Research and Clinical Oncology, 145(7), 1865–1875. 10.1007/s00432-019-02929-9 31123824 PMC11810420

[aphw12615-bib-0008] Cuevas, A. G. , O'Brien, K. , & Saha, S. (2019). Can patient‐centered communication reduce the effects of medical mistrust on patients' decision making? Health Psychology, 38(4), 325–333. 10.1037/hea0000721 30896219

[aphw12615-bib-0009] Curran, P. G. (2016). Methods for the detection of carelessly invalid responses in survey data. Journal of Experimental Social Psychology, 66, 4–19. 10.1016/j.jesp.2015.07.006

[aphw12615-bib-0010] DeSimone, J. A. , Harms, P. D. , & DeSimone, A. J. (2015). Best practice recommendations for data screening. Journal of Organizational Behavior, 36(2), 171–181. 10.1002/job.1962

[aphw12615-bib-0011] Douglas, K. M. , Uscinski, J. E. , Sutton, R. M. , Cichocka, A. , Nefes, T. , Ang, C. S. , & Deravi, F. (2019). Understanding conspiracy theories. Political Psychology, 40(S1), 3–35. 10.1111/pops.12568

[aphw12615-bib-0012] Finney Rutten, L. J. , Agunwamba, A. A. , Wilson, P. , Chawla, N. , Vieux, S. , Blanch‐Hartigan, D. , Arora, N. K. , Blake, K. , & Hesse, B. W. (2016). Cancer‐related information seeking among cancer survivors: Trends over a decade (2003–2013). Journal of Cancer Education, 31(2), 348–357. 10.1007/s13187-015-0802-7 25712202

[aphw12615-bib-0013] Fournier, V. , & Varet, F. (2024). Conspiracy beliefs and intention to use conventional, complementary and alternative medicines: Two vignette studies. British Journal of Health Psychology, 29(2), 333–350. 10.1111/bjhp.12702 37880094

[aphw12615-bib-0014] Franks, B. , Bangerter, A. , Bauer, M. W. , Hall, M. , & Noort, M. C. (2017). Beyond “monologicality”? Exploring conspiracist worldviews. Frontiers in Psychology, 8, 861. 10.3389/fpsyg.2017.00861 28676768 PMC5476781

[aphw12615-bib-0015] Galliford, N. , & Furnham, A. (2017). Individual difference factors and beliefs in medical and political conspiracy theories. Scandinavian Journal of Psychology, 58(5), 422–428. 10.1111/sjop.12382 28782805

[aphw12615-bib-0016] Granados Samayoa, J. A. , Moore, C. A. , Ruisch, B. C. , Boggs, S. T. , Ladanyi, J. T. , & Fazio, R. H. (2022). A gateway conspiracy? Belief in COVID‐19 conspiracy theories prospectively predicts greater conspiracist ideation. PLoS ONE, 17(10), e0275502. 10.1371/journal.pone.0275502 36288276 PMC9604008

[aphw12615-bib-0017] Green, D. W. , Horne, R. , & Shephard, E. A. (2013). Public perceptions of the risks, benefits and use of natural remedies, pharmaceutical medicines and personalised medicines. Complementary Therapies in Medicine, 21(5), 487–491. 10.1016/j.ctim.2013.07.007 24050584

[aphw12615-bib-0018] Grimes, D. R. (2022). The struggle against cancer misinformation. Cancer Discovery, 12(1), 26–30. 10.1158/2159-8290.CD-21-1468 34930788

[aphw12615-bib-0019] Hayes, A. F. (2022). Introduction to mediation, moderation, and conditional process analysis. A regression‐based approach (3rd ed.). Guilford Publications.

[aphw12615-bib-0020] Heley, K. , Chou, W.‐Y. S. , D'Angelo, H. , Senft Everson, N. , Muro, A. , Rohde, J. A. , & Gaysynsky, A. (2024). Mitigating health and science misinformation: A scoping review of literature from 2017 to 2022. Health Communication, 1–11. 10.1080/10410236.2024.2332817 38534199

[aphw12615-bib-0021] Huiart, L. , Bouhnik, A.‐D. , Rey, D. , Rousseau, F. , Retornaz, F. , Meresse, M. , Bendiane, M. K. , Viens, P. , & Giorgi, R. (2013). Complementary or alternative medicine as possible determinant of decreased persistence to aromatase inhibitor therapy among older women with non‐metastatic breast cancer. PLoS ONE, 8(12), e81677. 10.1371/journal.pone.0081677 24367488 PMC3867346

[aphw12615-bib-0022] Jacobs, J. M. , Ream, M. E. , Pensak, N. , Nisotel, L. E. , Fishbein, J. N. , MacDonald, J. J. , Buzaglo, J. , Lennes, I. T. , Safren, S. A. , Pirl, W. F. , Temel, J. S. , & Greer, J. A. (2019). Patient experiences with oral chemotherapy: Adherence, symptoms, and quality of life. Journal of the National Comprehensive Cancer Network, 17(3), 221–228. 10.6004/jnccn.2018.7098 30865917 PMC6626621

[aphw12615-bib-0023] Johnson, S. B. , Park, H. S. , Gross, C. P. , & Yu, J. B. (2018a). Complementary medicine, refusal of conventional cancer therapy, and survival among patients with curable cancers. JAMA Oncology, 4(10), 1375–1381. 10.1001/jamaoncol.2018.2487 30027204 PMC6233773

[aphw12615-bib-0024] Johnson, S. B. , Park, H. S. , Gross, C. P. , & Yu, J. B. (2018b). Use of alternative medicine for cancer and its impact on survival. JNCI Journal of the National Cancer Institute, 110(1), 121–124. 10.1093/jnci/djx145 28922780

[aphw12615-bib-0025] Johnson, S. B. , Parsons, M. , Dorff, T. , Moran, M. S. , Ward, J. H. , Cohen, S. A. , Akerley, W. , Bauman, J. , Hubbard, J. , Spratt, D. E. , Bylund, C. L. , Swire‐Thompson, B. , Onega, T. , Scherer, L. D. , Tward, J. , & Fagerlin, A. (2022). Cancer misinformation and harmful information on Facebook and other social media: A brief report. JNCI Journal of the National Cancer Institute, 114(7), 1036–1039. 10.1093/jnci/djab141 34291289 PMC9275772

[aphw12615-bib-0026] Jolley, D. , & Douglas, K. M. (2014). The social consequences of conspiracism: Exposure to conspiracy theories decreases intentions to engage in politics and to reduce one's carbon footprint. British Journal of Psychology, 105(1), 35–56. 10.1111/bjop.12018 24387095

[aphw12615-bib-0027] Kozyreva, A. , Lorenz‐Spreen, P. , Herzog, S. M. , Ecker, U. K. H. , Lewandowsky, S. , Hertwig, R. , Ali, A. , Bak‐Coleman, J. , Barzilai, S. , Basol, M. , Berinsky, A. J. , Betsch, C. , Cook, J. , Fazio, L. K. , Geers, M. , Guess, A. M. , Huang, H. , Larreguy, H. , Maertens, R. , … Wineburg, S. (2024). Toolbox of individual‐level interventions against online misinformation. Nature Human Behaviour, 8, 1044–1052. 10.1038/s41562-024-01881-0 38740990

[aphw12615-bib-0028] Krastev, S. , Krajden, O. , Vang, Z. M. , Juárez, F. P. , Solomonova, E. , Goldenberg, M. J. , Weinstock, D. , Smith, M. J. , Dervis, E. , Pilat, D. , & Gold, I. (2023). Institutional trust is a distinct construct related to vaccine hesitancy and refusal. BMC Public Health, 23, 2–17. 10.1186/s12889-023-17345-5 38082287 PMC10714562

[aphw12615-bib-0029] LaCroix, J. M. , Snyder, L. B. , Huedo‐Medina, T. B. , & Johnson, B. T. (2014). Effectiveness of mass media interventions for HIV prevention, 1986–2013: A meta‐analysis. JAIDS Journal of Acquired Immune Deficiency Syndromes, 66(Supplement 3), S329–S340. 10.1097/QAI.0000000000000230 25007204

[aphw12615-bib-0030] Lamberty, P. , & Imhoff, R. (2018). Powerful pharma and its marginalized alternatives?: Effects of individual differences in conspiracy mentality on attitudes toward medical approaches. Social Psychology, 49(5), 255–270. 10.1027/1864-9335/a000347

[aphw12615-bib-0031] Lantian, A. , Muller, D. , Nurra, C. , & Douglas, K. M. (2016). Measuring belief in conspiracy theories: Validation of a French and English single‐item scale. International Review of Social Psychology, 29(1), 1–14. 10.5334/irsp.8

[aphw12615-bib-0033] Lin, C. , Clark, R. , Tu, P. , Bosworth, H. B. , & Zullig, L. L. (2017). Breast cancer oral anti‐cancer medication adherence: A systematic review of psychosocial motivators and barriers. Breast Cancer Research and Treatment, 165(2), 247–260. 10.1007/s10549-017-4317-2 28573448

[aphw12615-bib-0034] Lin, H. , Garro, H. , Wernerfelt, N. , Shore, J. C. , Hughes, A. , Deisenroth, D. , Barr, N. , Berinsky, A. J. , Eckles, D. , Pennycook, G. , & Rand, D. G. (2024). Reducing misinformation sharing at scale using digital accuracy prompt ads. 10.31234/osf.io/u8anb

[aphw12615-bib-0035] Lleras De Frutos, M. , Casellas‐Grau, A. , Sumalla, E. C. , De Gracia, M. , Borràs, J. M. , & Ochoa Arnedo, C. (2020). A systematic and comprehensive review of internet use in cancer patients: Psychological factors. Psycho‐Oncology, 29(1), 6–16. 10.1002/pon.5194 31385400

[aphw12615-bib-0036] Lu, C. , Hu, B. , Bao, M.‐M. , Wang, C. , Bi, C. , & Ju, X.‐D. (2024). Can media literacy intervention improve fake news credibility assessment? A meta‐analysis. Cyberpsychology, Behavior, and Social Networking, 27(4), 240–252. 10.1089/cyber.2023.0324 38484319

[aphw12615-bib-0037] Marques, M. D. , Douglas, K. M. , & Jolley, D. (2022). Practical recommendations to communicate with patients about health‐related conspiracy theories. Medical Journal of Australia, 216(8), 381–384. 10.5694/mja2.51475 35430740 PMC9325074

[aphw12615-bib-0038] Martel, C. , & Rand, D. G. (2023). Misinformation warning labels are widely effective: A review of warning effects and their moderating features. Current Opinion in Psychology, 54, 101710. 10.1016/j.copsyc.2023.101710 37972523

[aphw12615-bib-0039] National Cancer Institute . (2023). Complementary and alternative medicine (CAM) . https://www.cancer.gov/about-cancer/treatment/cam

[aphw12615-bib-0040] National Center for Complementary and Integrative Health . (2021). Complementary, alternative, or integrative health: What's in a name? https://www.nccih.nih.gov/health/complementary-alternative-or-integrative-health-whats-in-a-name

[aphw12615-bib-0041] Natoli, E. E. , & Marques, M. D. (2021). The antidepressant hoax: Conspiracy theories decrease health‐seeking intentions. British Journal of Social Psychology, 60(3), 902–923. 10.1111/bjso.12426 33191598

[aphw12615-bib-0042] Oliveira, T. , Cardoso, N. D. O. , Machado, W. D. L. , Aragon Gonçalves, R. , Quinan, R. , Zorgi Salvador, E. , Almeida, C. , & Paes, A. (2024). Confronting misinformation related to health and the environment: A systematic review. Journal of Science Communication, 23(1). 10.22323/2.23010901

[aphw12615-bib-0044] Pennycook, G. , & Rand, D. G. (2022). Accuracy prompts are a replicable and generalizable approach for reducing the spread of misinformation. Nature Communications, 13(1), 2333. 10.1038/s41467-022-30073-5 PMC9051116 35484277

[aphw12615-bib-0045] Peterson, J. , Wilson, T. , Gruhl, J. , Davis, S. , Olsen, J. , Parsons, M. , Kann, B. , Fagerlin, A. , Watt, M. , & Johnson, S. (2022). Timing and motivations for alternative cancer therapy with insights from a crowdfunding platform: Cross‐sectional mixed methods study. JMIR Cancer, 8(2), e34183. 10.2196/34183 35671074 PMC9214612

[aphw12615-bib-0046] Pornpitakpan, C. (2004). The persuasiveness of source credibility: A critical review of five decades' evidence. Journal of Applied Social Psychology, 34(2), 243–281. 10.1111/j.1559-1816.2004.tb02547.x

[aphw12615-bib-0047] Rohrer, J. M. , Hünermund, P. , Arslan, R. C. , & Elson, M. (2022). That's a lot to process! Pitfalls of popular path models. Advances in Methods and Practices in Psychological Science, 5(2), 25152459221095827. 10.1177/25152459221095827

[aphw12615-bib-0048] Soveri, A. , Karlsson, L. C. , Antfolk, J. , Lindfelt, M. , & Lewandowsky, S. (2021). Unwillingness to engage in behaviors that protect against COVID‐19: The role of conspiracy beliefs, trust, and endorsement of complementary and alternative medicine. BMC Public Health, 21(1), 684. 10.1186/s12889-021-10643-w 33832446 PMC8027965

[aphw12615-bib-0050] Swire‐Thompson, B. , & Johnson, S. (2024). Cancer: A model topic for misinformation researchers. Current Opinion in Psychology, 56, 101775. 10.1016/j.copsyc.2023.101775 38101247 PMC10939853

[aphw12615-bib-0051] Toivonen, K. , Williamson, T. , Carlson, L. , Walker, L. , & Campbell, T. (2020). Potentially modifiable factors associated with adherence to adjuvant endocrine therapy among breast cancer survivors: A systematic review. Cancers, 13(1), 107. 10.3390/cancers13010107 33561076 PMC7794693

[aphw12615-bib-0052] Trella, C. , Sutton, R. M. , & Douglas, K. M. (2024). Semantic and causal relations between the conspiracy mentality and belief in conspiracy theories. Zeitschrift für Psychologie, 232(1), 7–17. 10.1027/2151-2604/a000545

[aphw12615-bib-0056] World Health Organization . (2024). Global cancer burden growing, amidst mounting need for services . https://www.who.int/news/item/01-02-2024-global-cancer-burden-growing-amidst-mounting-need-for-services PMC11115397 38438207

[aphw12615-bib-0057] Zhu, L. , Mou, W. , & Luo, P. (2024). Potential of large language models as tools against medical disinformation. JAMA Internal Medicine, 184(4), 450. 10.1001/jamainternmed.2024.0020 38407861

[aphw12615-bib-0059] Horne, R., Weinman, J., & Hankins, M. (1999). The beliefs about medicines questionnaire: The development and evaluation of a new method for assessing the cognitive representation of medication . *Psychology & Health, 14*(1), 1‑24. 10.1080/08870449908407311s

